# Impact of subcutaneous specific-allergen immunotherapy in the quality of life of brazilian moderate/severe atopic dermatitis patients

**DOI:** 10.1186/1939-4551-8-S1-A74

**Published:** 2015-04-08

**Authors:** Ana Julia Teixeira, Luciana Kase Tanno, Luciana Kase Tanno, Romero Kopke, Cintia Bassani, Veridiana Pereira-Aun, Wilson Aun, João Ferreira Mello

**Affiliations:** 1 Hospital Servidor Público Estadual De São Paulo, Brazil; 2 University of São Paulo, Brazil

## Background

Atopic dermatitis (AD) is known as having an important influence in the quality of life (Quol) of patients and their families, even though, the impact of the subcutaneous specific-allergen immunotherapy (SCIT) in this field has sparsely been accessed. This project aims to evaluate the effect of SCIT in the Quol of Brazilian patients with moderate/severe AD and their families.

## Methods

We analyzed 40 patients with diagnosis of moderate/severe AD based on the SCORAD and allergological work-up, under follow-up in the Allergy Department of our Hospital between 2012 to 2014. The cluster SCIT has been indicated based on the dust mite specific-IgE (*Dermatophagoides pteronyssinus* and/or *Blomia tropicalis*) and clinical relevance of these allergens. To access the quality of life of patients and their families, we used specific questionnaires previously validated to Brazilian Portuguese, the Infant’s Dermatitis Quality of Life Index (IDQoL) and Dermatitis Family Impact Questionnaire (DFI), and applied before and after 12 months of the use of SCIT. The Quol index scores were evaluated from 0 (best index of Quol) to 32 (worse index of Quol).

## Results

Of all 40 patients, 50% were women and the mean age was 12.4 years. The average index score of IDQoL before the immunotherapy was 13.59 and 7.7 after 12 months of the SCIT (P<0.001). Meanwhile, the average DFI was 13.54 in the first evaluation and 8.5 in the last evaluation (P<0.001). A positive correlation was observed between the severity of AD and IDQoL scores. The most important factors related to the decrease of the IDQoL were the improvement of pruritus (from 2.4 index to 0.9), quality of sleep (from 1.6 to 0.9) and feelings for having a cutaneous disease (from 1.3 to 0.7), the only domain we didn’t find significant difference was related to the impressions the patients have regarding the ongoing treatment (frequency of hospital visits). The major important domains of the DFI scores differences were the quality of the family’s sleep (from 1.0 index to 0.3), leisure (from 1.0 to 0.5) and costs (from 1.8 to 1.3). No significant difference was seen in the domain regarding the responsibility of the family on keeping the patient’s treatment.

## Conclusions

This study demonstrates that AD severity impaired the IDQoL as well as the DFI. The SCIT showed to be effective on increasing the Qol of AD patients and in their families, decreasing the index of the majority of both IDQol and IDF domains.

